# Supplementary material for: Aryl hydrocarbon receptor utilises cellular zinc signals to maintain the gut epithelial barrier
Source: Nat Commun. 2023 Sep 5;14:5431. doi: 10.1038/s41467-023-41168-y (PMC10480478; doi:10.1038/s41467-023-41168-y)
Supplement: Supplementary file 1 — Supplementary Information [file 41467_2023_41168_MOESM1_ESM.docx]

**Figure Legends for supplementary figures**

**
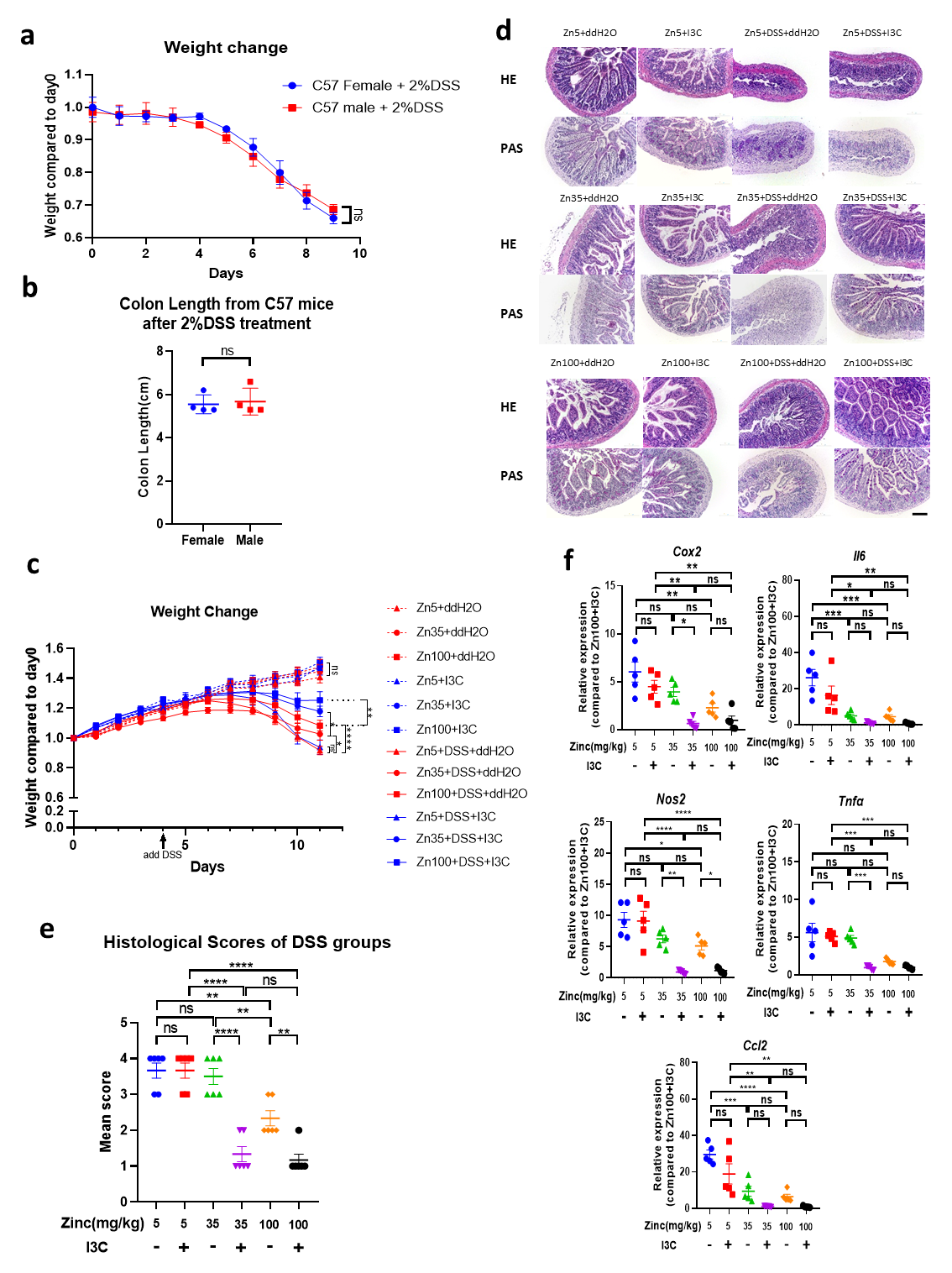
**

**Supplementary Figure 1: Effects of sex, zinc deficiency and I3C treatment in the DSS-induced IBD mouse model on body weight, histopathology of ileum, and expression of marker for epithelial barrier function**

(a) Weight change and (b) colon length of DSS-induced IBD model in sex-matched 6-week-old mice (n=4). (c) Mean daily weight change in female mice compared to Day 0 (n=6 for I3C groups and n=12 for ddH2O groups). Three-week-old C57BL/6J female mice were provided with diets with one of three zinc concentrations (5 mg/kg (Zinc5), 35 mg/kg (Zinc35) and 100 mg/kg (Zinc100) from Days 0-10 with (I3C) or without (ddH_2_O) I3C given by daily gavage. DSS was administered by the drinking water from Day 4 to Day 10. Mice were sacrificed on Day 11. (d) Histopathological changes in the ileum tissue of female mice examined by H&E and Periodic Acid Schiff^55^ staining (magnification, ×200). Scale bar, 200 μm (repeated for 6 times). (e) Histopathological scores of the ileum tissue in DSS treated female mice. (n=6) (f) RT-qPCR analysis of inflammatory cytokines (*Cox2, Il6, Tnfα and Nos2*) and Proinflammatory chemokine (*Ccl2*) in colon tissue samples from female mice (n=5). Scale bar, 200 μm. Representative data are means ± SEM and n=6 in each group. Animal treatments were repeated twice. Statistical analysis of the data was performed using 1-way ANOVA followed by Tukey’s multiple comparison tests. *p<0.05, **p<0.01, ***p<0.001, ****p<0.0001，ns not significant.


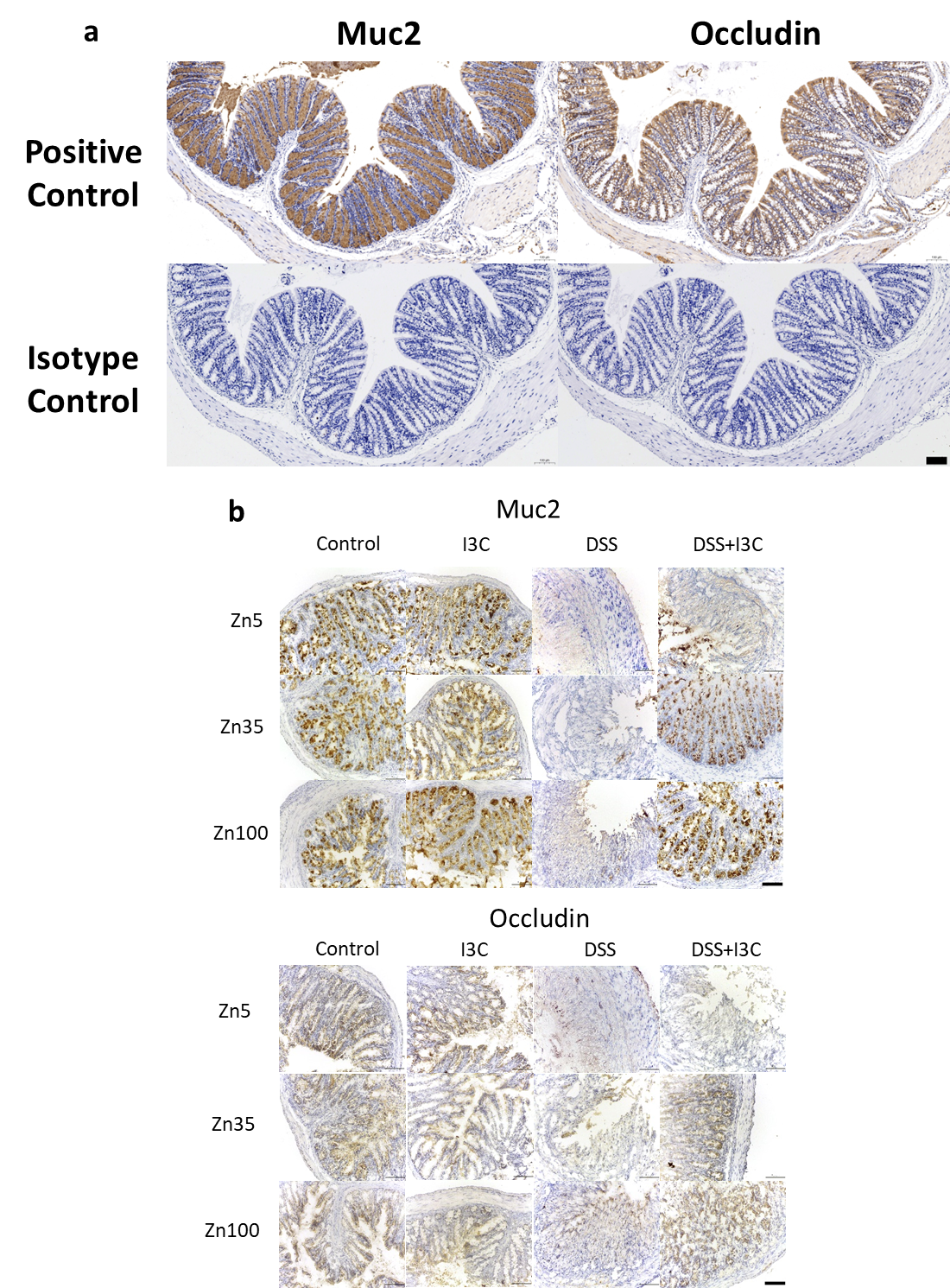


**Supplementary Figure 2: Representative immunocytochemistry images of MUC2 and Occludin from mouse intestine**

1. Representative positive control and isotype control of MUC2 and Occludin in colon tissues from WT mice (magnification, ×100). (b) Representative IHC images of MUC2 and Occludin expression in colon tissues (magnification, ×200) (repeated for 6 times).

**
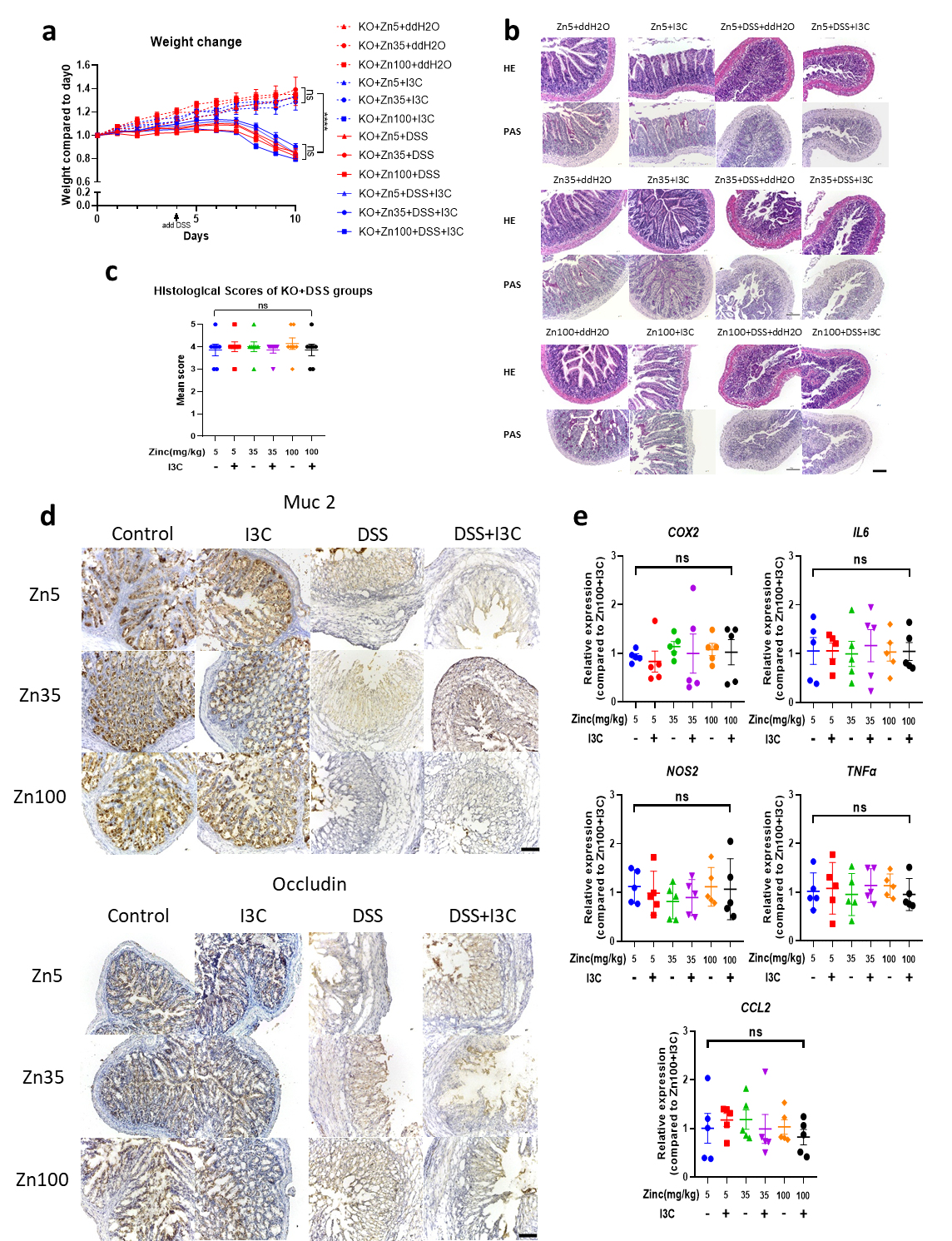
**

**Supplementary Figure 3: Effects of zinc deficiency and I3C treatment in the DSS-induced IBD model in Vil1-Ahr KO mice on body weight, histopathology of ileum, and expression of marker for epithelial barrier function**

Three-week-old villin^cre^ *Ahr*^fl/fl^ mice (KO) on C57BL/6J background were provided with diets with one of three zinc concentrations (5 mg/kg (Zinc5), 35 mg/kg (Zinc35) and 100 mg/kg (Zinc100) from Days 0-10 with (I3C) or without (ddH_2_O) I3C given by daily gavage. DSS was administered by the drinking water from Day 4 to Day10. Mice were sacrificed on Day 11. (a) Mean daily weight change compared to Day 0 (n=7 animals for DSS groups and n=6 animals for control groups). (b) Histopathological changes in the ileum tissue examined by H&E and Periodic Acid Schiff^55^ staining (magnification, ×200). Scale bar, 200 μm. (c) Histopathological scores of the ileum tissue in DSS treated mice (n=6). (d) Representative IHC images of MUC2 and Occludin expression in colon tissues (magnification, ×200). (e) RT-qPCR analysis of inflammatory cytokines (*Cox2, Il6, Tnfα and Nos2*) and Proinflammatory chemokine (*Ccl2*) in colon tissue samples (n=5). Scale bar, 200 μm. Animal treatments were repeated twice. Statistical analysis of the data was performed using 1-way ANOVA followed by Tukey’s multiple comparison test and data are means ±SEM. *p<0.05, **p<0.01, ***p<0.001, ****p<0.0001, ns not significant.


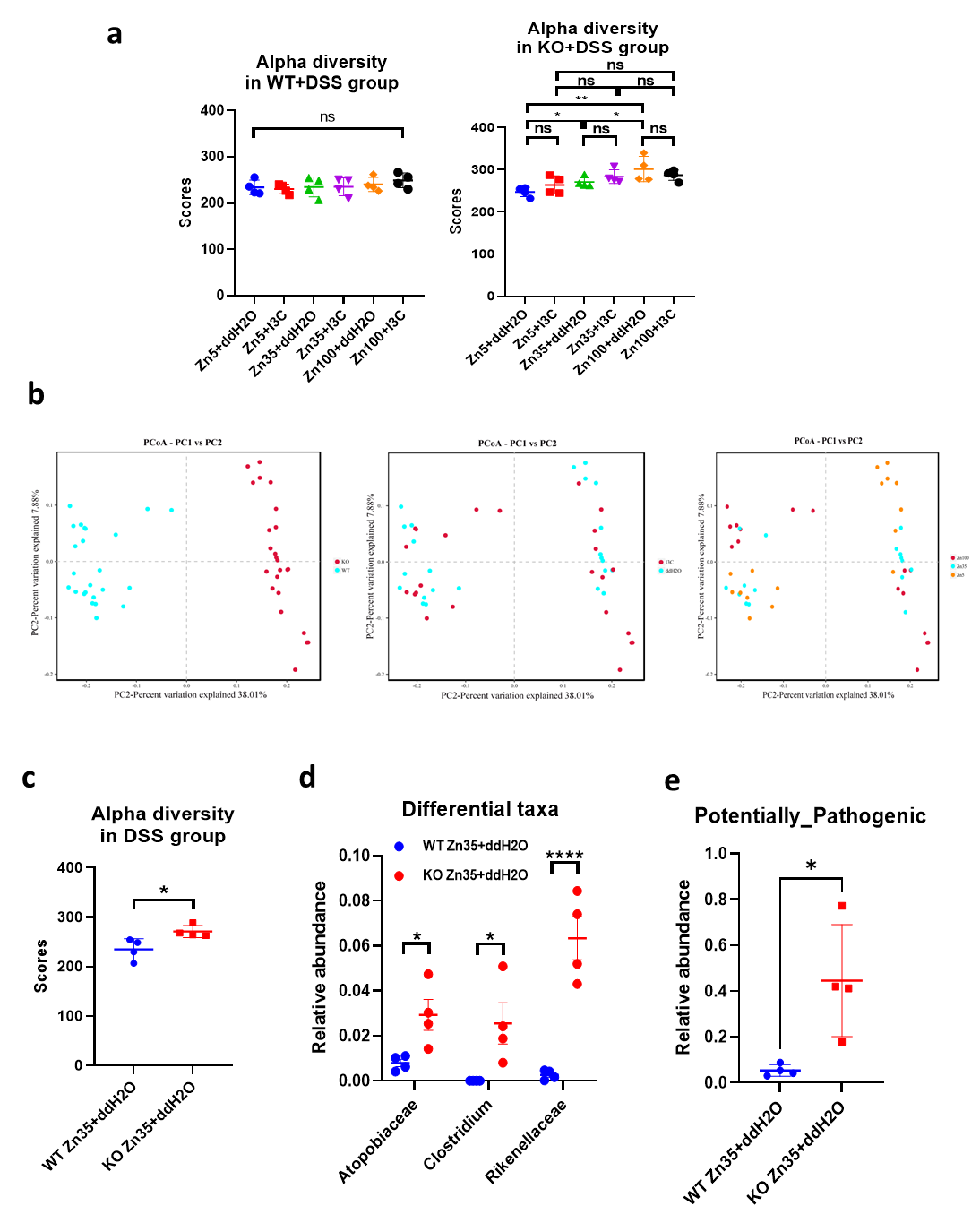


**Supplementary Figure 4: Analysis of intestinal microbiome in the DSS-treated WT and Vil1-AhR KO mice**

Three-week-old C57BL/6J (WT) mice and villin^cre^ *Ahr*^fl/fl^ mice (KO) on C57BL/6J background were provided with diets with one of three zinc concentrations (5 mg/kg (Zinc5), 35 mg/kg (Zinc35) and 100 mg/kg (Zinc100) from Days 0-10 with (I3C) or without (ddH_2_O) I3C given by daily gavage. DSS was administered by the drinking water from Day 4 to Day10. Mice were sacrificed on Day 11 and intestinal contents sampled for microbiome profiling. (a) Alpha diversity analysis of DSS-treated WT and villin^cre^Ahr^fl/fl^ groups. (b) Beta diversity analysis of WT and villin^cre^Ahr^fl/fl^ groups. Each plot is coloured according to the variables (WT or KO, I3C, Zn). PC1, PC2 represent the top two principal components that captures most of the differences in diversity. (c) Alpha diversity analysis between DSS-treated WT and villin^cre^AHR^fl/fl^ groups, both fed on the diet with intermediate zinc concentration (35 mg/kg (Zn35)) and not administered I3C (ddH_2_O). (d) The three most differing prokaryotic intestinal taxa between between DSS-treated WT and villin^cre^AHR^fl/fl^ groups, both fed on the diet with intermediate zinc concentration (35 mg/kg (Zn35)) and not administered I3C (ddH_2_O). (e) Potential pathogenicity prediction of DSS-treated WT and villin^cre^AHR^fl/fl^ groups, both fed on the diet with intermediate zinc concentration (35 mg/kg (Zn35)) and not administered I3C (ddH_2_O). Representative data are means ± SEM and n=4 in each group. Statistical analysis of the data was performed using 1-way ANOVA followed by Tukey’s multiple comparison test (Fig. a) and two-sided unpaired t-test (Fig. c, d, e). *p<0.05, **p<0.01, ***p<0.001, ****p<0.0001, ns not significant.


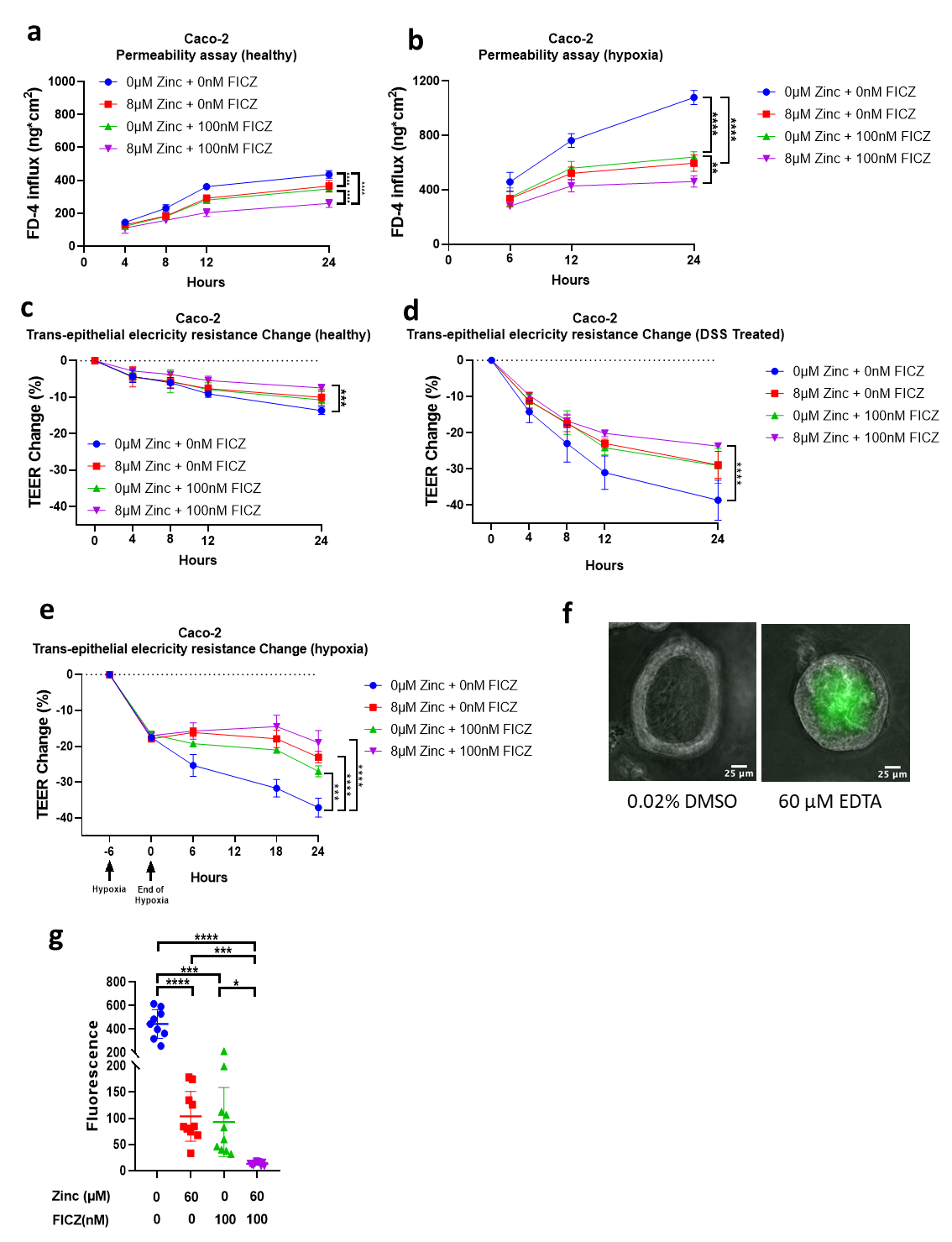


**Supplementary Figure 5: Additional evidence that combined treatment of AHR agonist FICZ and zinc promotes epithelial barrier function in Caco-2 cells and human ileum organoids**

(a-e) Caco-2 cells were grown to an epithelium in a Transwell^®^ system. At the start of the experiment, the medium in the apical compartment was replaced with MEM containing 0 or 8 µM zinc with or without 100 nM FICZ as indicated in the figure. (a,b) FITC-dextran 4000 (Merck) was added to the apical medium at t=0 and permeability was measured by sampling the medium in the basal compartment and measurement of FITC fluorescence over 24 hours. (a) The cells were left unchallenged to represent a healthy gut epithelium or (b) challenged with hypoxia for six hours prior to the start of the experiment and returned to atmospheric P_O2_ at t=0. (c-e) Changes in electrical resistance across Caco-2 cell epithelium kept in media with 0 or 8µM zinc with or without 100 nM FICZ. (A) The Caco-2 cell epithelium was unchallenged during the 24 h period representing a “Healthy” gut epithelium or challenged with (d) 3% DSS or (e) hypoxia for 6 h prior to the addition of zinc and/or FICZ. (f) FITC-Dextran leakage into lumen of human ileum organoids after 24 hours in 0.02 DMSO or 60 µM EDTA. (g) FITC-Dextran fluorescence in human ileum organoids challenged with 60 µM EDTA for 24 h in combination with 0 or 60 µM zinc with or without 100 nM.for 24 hours. Statistical analysis of the data was performed using 2-way ANOVA followed by Tukey’s multiple comparison tests. Caco-2 cell data are means ± SEM from three independent experiments. n=3 per group. Human ileum organoid data are means ± SEM from one experiment. n=10 per group. *p<0.05, **p<0.01, ***p<0.001, ****p<0.0001.

**Supplementary Figure 6: Combined treatment of AHR agonist FICZ inhibits activities of NF-κβ and calpains to improve tight junction protein abundance**

The stimulatory effects of zinc and FICZ co-treatment on expression of tight junction proteins and their respective transcripts are lost after inhibition of NF-κß or calpain. Abundance of transcripts for TJ proteins in Caco-2 cells treated with zinc and/or FICZ for 24 h after 4 h pre-treatment with (a) 10 μM NF-κß Blocker, QNZ (EVP4593) or (b) 50 μM Calpain Blocker, Calpeptin. Statistical analysis of the data was performed using two-sided unpaired t-tests. Data are means ± SEM from three independent experiments. n=3 per group. *p<0.05, **p<0.01, ***p<0.001, ****p<0.0001, ns not significant.

**Supplementary Figure 7: Western Blots showing whole blots of ZO-1 and occludin**

Whole Western Blots corresponding to data shown in Figure 3.

**Supplementary Figure 8: Western Blots showing whole blots for phosphorylation of NF-κβ subunits and whole blots for ZO-1 and OCLN**

Whole Western Blots for IκBα^ser32/36^, P65^ser536^, total IκBα, and P65, and ZO-1 and OCLN corresponding to data shown in Figure 4 and 5.

**Supplementary Figure 9: Verification of intestinal epithelium deletion of *Ahr* in mice**

*Ahr* floxed mice and *Vil1*-Cre mice were crossed for obtaining specific deletion of *Ahr* in villus epithelial cells of the small and large intestines (*villin*^cre^*Ahr*^fl/fl^). (a). Genotyping results of *Ahr* loxp (+/+) *Vil1*-cre (+/-) mice. (b). Western Blotting results of AHR in intestinal epithelial cells from C57BL/6J and *Ahr* loxp (+/+) Vil1-cre (+/-) mice. (c). Western Blotting results of AHR in liver tissue from C57BL/6J and *Ahr* loxp (+/+) *Vil1*-cre (+/-) mice. All Ahr loxp (+/+) Vil1-cre (+/-) mice are tested by genotyping and western blotting results are only performed in the first generation.

**Supplementary Table 1: qPCR primers used in AHR ChIP-qPCR and location of amplicons and the respective genes.** Genomic locations of genes and PCR fragments are from ENSEMBL human genome assembly GRCh38.p13

**Supplementary Table 2: Primers for qPCR for gene expression analysis.** Probe refers to the identifiers in the Universal Probe Library (UPL).

| ***Gene*** | ***Forward Primer*** | ***Reverse Primer*** | ***Probe*** |
| --- | --- | --- | --- |
| *human* |  |  |  |
| *MTF1* | *AAGGGTGTGGTTTCCATCAA* | *GATCCACAAAGACCCCAGTG* | *#44* |
| *MT1A* | *CTTGGGATCTCCAACCTCAC* | *GCATTTGCAGGAGCCAGT* | *#68* |
| *SLC39A2* | *GAACAGATCAGCAAGTGAGAGAAA* | *AGCTCTCCATAGGGATACTCCA* | *#09* |
| *SLC39A4* | *CCTCTTCCTGCTGCACAAC* | *CATCCTCGTACAGGGACAGC* | *#03* |
| *SLC39A6* | *ACTGGCCGTTGGGACTTT* | *ATGGTGGTGACTTGCATGAG* | *#09* |
| *SLC39A7* | *ATGGAGGCTATGGGGAGTCT* | *GGGGATAAGGAAGAGGACAAA* | *#01* |
| *SLC39A10* | *TGAATACACGATTTGGTGCAG* | *TTGTGTGCATATGTACCTTCATTTC* | *#55* |
| *MUC2* | *ACCCACCAGCACACAGAGTA* | *GGGGTTGGGGTTACCGTAT* | *#09* |
| *CLDN1* | *CCTATGACCCCAGTCAATGC* | *ACAGCAAAGTAGGGCACCTC* | *#08* |
| *CLDN3* | *AACCTGCATGGACTGTGAAA* | *GGTCAAGTATTGGCGGTCAC* | *#50* |
| *CLDN4* | *GGGACTGGGCAGAGACTG* | *TTGGGAAGTTGTCCGAGTG* | *#08* |
| *OCLN* | *AGGAACCGAGAGCCAGGT* | *TGAGCAATGCCCTTTAGCTT* | *#84* |
| *ZO-1* | *TGCATGATGATCGTCTGTCC* | *AAGTGTGTCTACTGTCCGTGCTAT* | *#01* |
| *GAPDH* | *AGCCACATCGCTCAGACAC* | *GCCCAATACGACCAAATCC* | *#60* |
| *UBC* | *GGAAGGCATTCCTCCTGAT* | *CCCACCTCTGAGACGGAGTA* | *#11* |
| *Beta-Actin* | *AGAGCTACGAGCTGCCTGAC* | *CGTGGATGCCACAGGACT* | *#09* |
| *mouse* |  |  |  |
| *Mtf1* | *CCAAGAGACTAGTTGGCAGCA* | *GGTGGGACCAAGATCACCT* | *#10* |
| *Slc39A4* | *CAGCTACTGCAGAAGATTGAGG* | *TCCAGCAGTTGGGGAAGAT* | *#07* |
| *Slc39A6* | *CCAGTCCCTTCGGACCTC* | *CTGTGGCCATTGCACCTT* | *#70* |
| *Slc39A7* | *GGATTTTGCCATCCTGGTC* | *TTGCAGTCACGAGTTGCAG* | *#71* |
| *Slc39A10* | *TTTCAGATCATAAGTTAAACAGCACA* | *CCGAGTCATCCGTTCCAG* | *#89* |
| *Ubc* | *GACCAGCAGCAGGCTGATCTT* | *CCTCTGAGGCGAAGGACTAA* | *#11* |
| *Ccl2* | *GCTACAAGAGGATCACCAGCAG* | *GTCTGGACCCATTCCTTCTTGG* | *origene* |
| *Cox2* | *GCGACATACTCAAGCAGGAGCA* | *AGTGGTAACCGCTCAGGTGTTG* | *origene* |
| *Nos2* | *GAGACAGGGAAGTCTGAAGCAC* | *CCAGCAGTAGTTGCTCCTCTTC* | *origene* |
| *Il6* | *TACCACTTCACAAGTCGGAGGC* | *CTGCAAGTGCATCATCGTTGTTC* | *origene* |
| *Tnfα* | *GGTGCCTATGTCTCAGCCTCTT* | *GCCATAGAACTGATGAGAGGGAG* | *origene* |

**Supplementary Table 3: Antibodies use in Western Blot of cell line and organoids with associated information on dilutions and blocking agent used.**

| **Antibody Name** | **Company and Cat No.** | **Dilution** | **Blocking agent** |
| --- | --- | --- | --- |
| GAPDH | Sigma-Aldrich, MAB374 | 1:15,000 | 5% skim milk |
| GAPDH | Cell Signalling Technology, 2118 | 1:2500 | 5% skim milk |
| ZO-1 | Cell Signalling Technology, 13663 | 1:800 | 5% skim milk |
| Occludin | Santa Cruz Biotechnology, sc-133256 | 1:200 | 5% skim milk |
| Occludin | Santa Cruz Biotechnology, sc-133256 | 1:100 | 5 % normal goat serum |
| Occludin | Servicebio, GB111401 | 1:500, | 3% BSA |
| Muc2 | Santa Cruz Biotechnology, sc-515032 AF488 | 1:100 | 5 % normal goat serum |
| Muc2 | Servicebio, GB11344, | 1:500 | 3% BSA |
| Claudin1 | Invitrogen, 51-9000 | 1:250 | 5% skim milk |
| Claudin3 | Invitrogen, 34-1700 | 1:250 | 5% skim milk |
| Claudin4 | Invitrogen,32-9400 | 1:250 | 5% skim milk |
| Phospho-NF-κB p65 | Cell Signalling Technology, 3033 | 1:1000 | 5% BSA |
| NF-κB p65 | Cell Signalling Technology, 8242 | 1:1000 | 5% skim milk |
| Phospho-IκBα | Cell Signalling Technology, 9246 | 1:1000 | 5% BSA |
| IκBα | Cell Signalling Technology, 4814 | 1:1000 | 5% skim milk |
| AhR | Enzo Life Sciences, BML-SA210-0100 | 1:2000 | 3% BSA |
| Beta tubulin | Abcam,ab6046 | 1:5000 | 3% BSA |
| Anti-Mouse IgG | Bio-rad, 1705047 | 1:10,000 | 5% skim milk |
| Anti-Rabbit IgG | Santa Cruz Biotechnology, sc-2357 | 1:4000 | 5% skim milk |
| Goat anti-Mouse IgG | Invitrogen, A-11031 | 1: 250 | 5 % normal goat serum |
| Goat Anti-rabbit  IgG | Servicebio, GB1213 | 1: 200 | 3% BSA |

**Supplementary Table 4: Composition of 5 ppm Zinc, 35 ppm Zinc and 100 ppm Zinc diets.**

| **Product#** | **5 ppm Zinc** | | **35 ppm Zinc** | | **100 ppm Zinc** | |
| --- | --- | --- | --- | --- | --- | --- |
| **Ingredient** | **gm** | **kcal** | **gm** | **kcal** | **gm** | **kcal** |
| Egg Whites, Dried | 200 | 800 | 200 | 800 | 200 | 800 |
| Corn Starch | 150 | 600 | 150 | 600 | 150 | 600 |
| Sucrose | 502.38 | 2010 | 502.38 | 2010 | 502.41 | 2010 |
| Cellulose, BW200 | 50 | 0 | 50 | 0 | 50 | 0 |
| Corn Oil | 50 | 450 | 50 | 450 | 50 | 450 |
| Mineral Mix S19401 (No Zn) | 35.0 | 0 | 35.0 | 0 | 0.0 | 0 |
| Mineral Mix S10001 | 0.0 | 0 | 0.0 | 0 | 35.0 | 0 |
| Zinc Carbonate, 52.1% Zinc | 0.0080 | 0 | 0.0660 | 0 | 0.105 | 0 |
| Vitamin Mix V19401 | 10.4 | 40 | 10.4 | 40 | 10.4 | 40 |
| Choline Bitartrate | 2 | 0 | 2 | 0 | 2 | 0 |
| Total | 999.79 | 3900 | 999.85 | 3900 | 999.92 | 3900 |
