# Supplementary material for: Aryl hydrocarbon receptor utilises cellular zinc signals to maintain the gut epithelial barrier
Source: Nat Commun. 2023 Sep 5;14:5431. doi: 10.1038/s41467-023-41168-y (PMC10480478; doi:10.1038/s41467-023-41168-y)
Supplement: Supplementary file 3 — Reporting Summary [file 41467_2023_41168_MOESM3_ESM.pdf]

nature portfolio

Corresponding author(s): *Double-blind peer review submissions: write DBPR and your manuscript number here instead of author names.*

Last updated by author(s): *YYYY-MM-DD*

Reporting Summary

Nature Portfolio wishes to improve the reproducibility of the work that we publish. This form provides structure for consistency and transparency in reporting. For further information on Nature Portfolio policies, see our Editorial Policies and the Editorial Policy Checklist.

Statistics

For all statistical analyses, confirm that the following items are present in the figure legend, table legend, main text, or Methods section.

n/a	Confirmed	
☐	☒	The exact sample size (*n*) for each experimental group/condition, given as a discrete number and unit of measurement
☐	☒	A statement on whether measurements were taken from distinct samples or whether the same sample was measured repeatedly
☐	☒	The statistical test(s) used AND whether they are one- or two-sided<br>*Only common tests should be described solely by name; describe more complex techniques in the Methods section.*
☒	☐	A description of all covariates tested
☒	☐	A description of any assumptions or corrections, such as tests of normality and adjustment for multiple comparisons
☐	☒	A full description of the statistical parameters including central tendency (e.g. means) or other basic estimates (e.g. regression coefficient) AND variation (e.g. standard deviation) or associated estimates of uncertainty (e.g. confidence intervals)
☐	☒	For null hypothesis testing, the test statistic (e.g. *F*, *t*, *r*) with confidence intervals, effect sizes, degrees of freedom and *P* value noted<br>*Give P values as exact values whenever suitable.*
☒	☐	For Bayesian analysis, information on the choice of priors and Markov chain Monte Carlo settings
☒	☐	For hierarchical and complex designs, identification of the appropriate level for tests and full reporting of outcomes
☒	☐	Estimates of effect sizes (e.g. Cohen's *d*, Pearson's *r*), indicating how they were calculated

Our web collection on statistics for biologists contains articles on many of the points above.

Software and code

Policy information about availability of computer code

Data collection	High-throughput sequencing analysis of bacterial 16S rDNA genes was performed based on the Illumina Novaseq at Biomarker Technologies Corporation (Beijing, China).
Data analysis	According to the relationship between the overlap and paired-end reads, raw tags were obtained from the splicing sequence using Trimmomatic (version 0.33). Then, Cutadapt (version 1.9.1) was used to obtain clean tags and effective tags, respectively. Clustering of the effective tags into operational taxonomic units (OTUs) at 97% similarity was performed using USEARCH (version 10.0). Then, OTUs were annotated using RDP Classifier (version 2.2, at 0.8 confidence threshold) based on the Silva taxonomy database. Finally, alpha diversity, beta diversity and phenotype prediction were performed using QIIME2, QIIME and Bugbase, respectively. The statistical analyses were performed using GraphPad Prism 9.0 software.

For manuscripts utilizing custom algorithms or software that are central to the research but not yet described in published literature, software must be made available to editors and reviewers. We strongly encourage code deposition in a community repository (e.g. GitHub). See the Nature Portfolio guidelines for submitting code & software for further information.

Data

Policy information about availability of data

All manuscripts must include a data availability statement. This statement should provide the following information, where applicable:

- Accession codes, unique identifiers, or web links for publicly available datasets
- A description of any restrictions on data availability
- For clinical datasets or third party data, please ensure that the statement adheres to our policy

The sequencing data have been deposited in the NCBI Sequence Read Archive (SRA) database (http://www.ncbi.nlm.nih.gov/sra) with the accession numbers PRJNA945597.

Human research participants

Policy information about studies involving human research participants and Sex and Gender in Research.

Reporting on sex and gender	n/a
Population characteristics	n/a
Recruitment	n/a
Ethics oversight	n/a

Note that full information on the approval of the study protocol must also be provided in the manuscript.

Field-specific reporting

Please select the one below that is the best fit for your research. If you are not sure, read the appropriate sections before making your selection.

☒ Life sciences ☐ Behavioural & social sciences ☐ Ecological, evolutionary & environmental sciences

For a reference copy of the document with all sections, see nature.com/documents/nr-reporting-summary-flat.pdf

Life sciences study design

All studies must disclose on these points even when the disclosure is negative.

Sample size	The sample size of the in vitro experiment was generally 3-4 repeated experiments, and that of the in vivo experiment was 6~7 for the high intra-group difference.
Data exclusions	No data were excluded from the analysis.
Replication	The in vitro experiments were repeated 3 times and the in vivo experiments were repeated twice.
Randomization	No formal randomization techniques was used.
Blinding	Blinding was not used in the experiment for the administration and statistics of a specific group of mice were involved.

Reporting for specific materials, systems and methods

We require information from authors about some types of materials, experimental systems and methods used in many studies. Here, indicate whether each material, system or method listed is relevant to your study. If you are not sure if a list item applies to your research, read the appropriate section before selecting a response.

Materials & experimental systems

n/a	Involved in the study
☐	☒ Antibodies
☐	☒ Eukaryotic cell lines
☒	☐ Palaeontology and archaeology
☐	☒ Animals and other organisms
☒	☐ Clinical data
☒	☐ Dual use research of concern

Methods

n/a	Involved in the study
☒	☐ ChIP-seq
☒	☐ Flow cytometry
☒	☐ MRI-based neuroimaging

Antibodies

Antibodies used

Antibody Name Company and Cat No.
GAPDH Sigma-Aldrich, MAB374
GAPDH Cell Signalling Technology, 2118
ZO-1 Cell Signalling Technology, 13663
Occludin Santa Cruz Biotechnology, sc-133256
Occludin Santa Cruz Biotechnology, sc-133256
Occludin Servicebio, GB111401
Muc2 Santa Cruz Biotechnology, sc-515032 AF488
Muc2 Servicebio, GB11344,
Claudin1 Invitrogen, 51-9000
Claudin3 Invitrogen, 34-1700
Claudin4 Invitrogen,32-9400
Phospho-NF-κB p65 Cell Signalling Technology, 3033
NF-κB p65 Cell Signalling Technology, 8242
Phospho-IκBα Cell Signalling Technology, 9246
IκBα Cell Signalling Technology, 4814
Anti-Mouse IgG Bio-rad, 1705047
Anti-Rabbit IgG Santa Cruz Biotechnology, sc-2357
Goat anti-Mouse IgG Invitrogen, A-11031
Goat Anti-rabbit IgG Servicebio, GB1213

Validation

each antibody was validated in the preliminary experiment to confirm the concentration and specific binding, as well as the the suitable blocking reagent.

Antibody Name Company and Cat No. Dilution Blocking agent
GAPDH Sigma-Aldrich, MAB374 1:20,000 5% skim milk
GAPDH Cell Signalling Technology, 2118 1:2500 5% skim milk
ZO-1 Cell Signalling Technology, 13663 1:800 5% skim milk
Occludin Santa Cruz Biotechnology, sc-133256 1:200 5% skim milk
Occludin Santa Cruz Biotechnology, sc-133256 1:100 5 % normal goat serum
Occludin Servicebio, GB111401 1:500, 3% BSA
Muc2 Santa Cruz Biotechnology, sc-515032 AF488 1:100 5 % normal goat serum
Muc2 Servicebio, GB11344, 1:500 3% BSA
Claudin1 Invitrogen, 51-9000 1:250 5% skim milk
Claudin3 Invitrogen, 34-1700 1:250 5% skim milk
Claudin4 Invitrogen,32-9400 1:250 5% skim milk
Phospho-NF-κB p65 Cell Signalling Technology, 3033 1:1000 5% BSA
NF-κB p65 Cell Signalling Technology, 8242 1:1000 5% skim milk
Phospho-IκBα Cell Signalling Technology, 9246 1:1000 5% BSA
IκBα Cell Signalling Technology, 4814 1:1000 5% skim milk
Anti-Mouse IgG Bio-rad, 1705047 1:10,000 5% skim milk
Anti-Rabbit IgG Santa Cruz Biotechnology, sc-2357 1:4000 5% skim milk
Goat anti-Mouse IgG Invitrogen, A-11031 1: 250 5 % normal goat serum
Goat Anti-rabbit IgG Servicebio, GB1213 1: 200 3% BSA

Eukaryotic cell lines

Policy information about cell lines and Sex and Gender in Research

Cell line source(s)

This specific organoid line was isolated from biopsies of a 29-year-old female patient from St Thomas NHS Foundation Trust in 2018. No regular medication record.

Caco-2 [Caco2] are epithelial cells isolated from colon tissue derived from a 72-year-old, White, male with colorectal adenocarcinoma.

Authentication

Human organoid line was derived from ileum. 3D structure was shown in the microscopy. Lyz measurement and image were confirm as the ileum organoid line.

	Caco-2 cell line were gifted from the colleague who bought from ATCC.
Mycoplasma contamination	Mycoplasma was detected every three months and no Mycoplasma contamination
Commonly misidentified lines (See ICLAC register)	n/a

Animals and other research organisms

Policy information about studies involving animals; ARRIVE guidelines recommended for reporting animal research, and Sex and Gender in Research

Laboratory animals	Three-week-old C57BL/6J mice and three-week-old villin-cre Ahr(fl/fl) mice
Wild animals	n/a
Reporting on sex	Only female mice invovled in this experiment for the sex of mice did not affect the phenotype of DSS-induced IBD model. And single sex was used to reduce experimental variables.
Field-collected samples	n/a
Ethics oversight	All experiments complied with the relevant laws and institutional guidelines, as overseen by the Animal Studies Committee of the Children's Hospital of Fudan University.

Note that full information on the approval of the study protocol must also be provided in the manuscript.
